# Using Integrin α_v_β_6_-Targeted Positron Emission Tomography Imaging to Longitudinally Monitor Radiation-Induced Pulmonary Fibrosis In Vivo

**DOI:** 10.1016/j.ijrobp.2024.08.034

**Published:** 2024-09-14

**Authors:** William C.Y. Lo, Cristian W. Villas Boas, Truc T. Huynh, Amanda Klaas, Felicia Grogan, Lori Strong, Pamela Samson, Clifford G. Robinson, Buck E. Rogers, Carmen Bergom

**Affiliations:** * Department of Radiation Oncology, Washington University School of Medicine, St. Louis, Missouri; † Department of Chemistry, Washington University in St Louis, St Louis, Missouri

## Abstract

**Purpose:**

Radiation-induced pulmonary fibrosis (RIPF) is a potentially serious and disabling late complication of radiation therapy. Monitoring RIPF progression is challenging due to the absence of early detection tools and the difficulty in distinguishing RIPF from other lung diseases using standard imaging methods. In the lungs, integrin *α*v*β*6 is crucial in the development of RIPF, acting as a significant activator of transforming growth factor *β* after radiation injury. This study aimed to investigate integrin *α*_v_*β*_6_–targeted positron emission tomography (PET) imaging ([^64^Cu]Cu-*α*_v_*β*_6_-BP) to study RIPF development in vivo.

**Methods and Materials:**

We used a focal RIPF model (70 Gy delivered focally to a 3 mm spot in the lung) and a whole lung RIPF model (14 Gy delivered to the whole lung) in adult C57BL/6J mice. Small animal PET/computed tomography images were acquired 1 hour postinjection of 11.1 MBq of [^64^Cu]Cu-*α*_v_*β*_6_-BP. Animals were imaged for 8 weeks in the focal RIPF model and 6 months in the whole lung RIPF model. Immunohistochemistry for integrin *α*_v_*β*_6_ and trichrome staining were performed.

**Results:**

In the focal RIPF model, there was focal uptake of [^64^Cu]Cu-*α*_v_*β*_6_-BP in the irradiated region at week 4 that progressively increased at weeks 6 and 8. In the whole lung RIPF model, minimal uptake of the probe was observed at 4 months post–radiation therapy, which significantly increased at months 5 and 6. Expression of integrin *α*_v_*β*_6_ was validated histologically by immunohistochemistry in both models.

**Conclusions:**

Integrin *α*_v_*β*_6_–targeted PET imaging using [^64^Cu]Cu-*α*_v_*β*_6_-BP can serve as a useful tool to identify RIPF in vivo.

## Introduction

Radiation-induced pulmonary fibrosis (RIPF) is a potentially debilitating late complication from radiation therapy (RT), and it is associated with a poor prognosis in cancer survivors.^1-4^ RIPF is a particularly challenging dose-limiting normal tissue toxicity in cases such as pediatric patients undergoing total body irradiation for hematological malignancies, as well as patients with lung cancer, especially those with extensive disease burden. Its incidence correlates with the volume of the lung treated and the radiation dose delivered. Currently, clinical monitoring of RIPF progression remains challenging given the lack of imaging tools that can reliably detect RIPF at an early stage and the difficulty distinguishing RIPF from other pulmonary pathologies using conventional imaging tools. Therefore, the ability to accurately identify RIPF development at an early stage and discern it from other pathologies has significant clinical implications.

In the lung, the integrin cell surface receptors play a key role in the pathogenesis of RIPF. In particular, integrin *α*_v_*β*_6_ is expressed at a low level in the alveolar epithelium at baseline, and its expression is significantly upregulated on radiation damage.^5^ Integrin *α*_v_*β*_6_ is a major transforming growth factor-*β* (TGF-*β*) activator in the lung, critical to the pathogenesis of pulmonary fibrosis.^5-8^ TFG-*β* is secreted in the form of inactive complexes with latency-associated peptides (LAPs).^9^ Integrin *α*_v_*β*_6_ interacts with the linear arginine-glycine-aspartic acid (RDG) motif of LAP, activating TFG-*β*, specifically TFG-*β*1, which is most involved in fibrogenesis.^10^ In addition, mice lacking integrin *α*_v_*β*_6_ do not develop fibrosis, demonstrating the key role of integrin *α*_v_*β*_6_ in fibrogenesis.^11^

The radiotracer [^18^F]F-A20FMDV2 targeting integrin *α*_v_*β*_6_ was developed in 2007,^12^ and it was optimized by the addition of polyethylene glycol 28 (PEG_28_) on both sides in 2015, which was shown to improve tumor uptake of the radiotracer in mice bearing BxPC-3 xenografts.^13^ Later, in 2019, there was a first-in-human positron emission tomography/computed tomography (PET/CT) study of this probe with a K16R substitution in patients with metastatic carcinoma.^14^ To demonstrate the role of integrin *α*_v_*β*_6_ in pulmonary fibrosis, integrin *α*_v_*β*_6_-based single-photon emission computed tomography (SPECT)/CT imaging has been examined in preclinical bleomycin-induced pulmonary fibrosis models using an ^111^In-labeled A20FMDV2 peptide, which shows highly selective binding to integrin *α*_v_*β*_6_.^7^ The study demonstrated increased ^111^In-labeled A20FMDV2 uptake in bleomycin-treated mice compared with control mice, and attenuated uptake with an integrin *α*_v_*β*_6_–blocking antibody. Saleem et al^15^ performed PET/CT with [18F]-FBA-A20FMDV2 in non-small cell lung cancer patients within 6 months of completion of their radiation therapy and found that regional tracer uptake correlated with the dose per fraction and the biologically equivalent dose (BED), supporting a role of *α*_v_*β*_6_ induction of lung fibrosis after radiation. Lukey et al^6^ studied clinical quantification of integrin avb6 using [^18^F]F-A20FMDV2 PET imaging in patients with idiopathic pulmonary fibrosis and demonstrated significantly increased uptake in the lung of these patients (predominately confined to fibrotic areas) compared with healthy volunteers. Kimura et al^16^ further evaluated the use of integrin *α*_v_*β*_6_ cystine-knot PET tracers to image patients with idiopathic pulmonary fibrosis and multiple cancer types. Interestingly, a high level of integrin *α*_v_*β*_6_ expression is associated with poor overall survival in patients with idiopathic pulmonary fibrosis,^17^ suggesting that integrin *α*_v_*β*_6_ level can potentially serve as an important prognostic marker in patients. Finally, an alternative approach for imaging pulmonary fibrosis in preclinical models involves the use of type I collagen-targeted ^68^Ga-CBP8.^18^ Although this technique enables direct quantification of type I collagen, it does not enable the detection of disease prior to fibrosis development. However, to our knowledge, integrin *α*_v_*β*_6_-based PET imaging has not been studied in the context of pre-clinical RIPF models.

In this study, we investigated an integrin *α*_v_*β*_6_-targeted PET imaging probe ^64^[Cu]Cu-*α*_v_*β*_6_-BP (synthesized with the A20FMDV2 peptide with the K16R substitution^14,19^) to study the evolution of RIPF development in 2 preclinical models: (1) a focal ablative RIPF model^20^ (that leads to significantly accelerated development of fibrosis over 8 weeks) and (2) a whole lung RIPF model^5^ (with fibrosis development over 6 months). This study hypothesized that integrin *α*_v_*β*_6_-targeted PET imaging enables longitudinal monitoring of RIPF in vivo by serving as an early molecular imaging biomarker that correlates with the severity of pulmonary fibrosis.

## Methods and Materials

### Focal RIPF model and whole lung RIPF model

All animal experiments were performed according to the Guidelines for Care and Use of Animals established by the Division of Comparative Medicine and the Animal Studies Committee at Washington University in St. Louis under protocols #20-0214 and #20-0266.

Using the small animal radiation research platform (SARRP; XStrahl), the left lungs of 4 C57BL/6J mice (female, aged 10 weeks) were irradiated focally at 70 Gy using a 3 × 3 mm beam collimator with a 1:1 AP:PA configuration (225 kV, 13 mA, 0.15 mm Cu filter, 3.67 Gy/min; Fig. E1). The dose, collimator size, and imaging time points were chosen based on a prior study investigating small animal models for stereotactic body RT, which demonstrated significantly accelerated development of fibrosis in 4 to 8 weeks.^20,21^ In addition, 4 sham-irradiated animals served as control. Small animal PET/CT imaging was performed at weeks 4, 6, and 8 longitudinally. In the whole lung RIPF model, the right lungs of 4 C57BL/6J mice per time point (female, aged 10 weeks) were irradiated with a single 14 Gy fraction using a 10 × 10 mm beam collimator (1:1 AP:PA configuration; Fig. E2) and imaging was performed from months 4 to 6 (imaging time points were selected based on a prior study demonstrating the upregulation of integrin *α*_v_*β*_6_ expression at ~18-20 weeks after 14 Gy was delivered to the thorax^5^). In addition, 4 sham-irradiated animals served as control.

### Radiochemical synthesis of [^64^Cu]Cu-α_v_β_6_-BP

The peptide DOTA-PEG_28_-NAVPNLRGDLQVLAQR-VART-PEG_28_, herein abbreviated as *α*_v_*β*_6_-binding peptide (*α*_v_*β*_6_-BP), was synthesized and characterized by AnaSpec. DOTA is the chelator, 1,4,7,10-tetraazacyclododecane-1,4,7,10-tetraacetic acid. Stock solutions of the BP were prepared with High performance liquid chromatography-grade water at 1 nmol/*μ*L, fractionated in 10 *μ*L, and stored at −20°C until use. Quality control was performed with Radio-TLCs Whatman 60 Å silica gel thin-layer chromatography (TLC) plates and analyzed using a Bioscan 200 imaging scanner (Bioscan, Inc. All solvents and reagents were purchased from Sigma-Aldrich or Fisher Scientific and used as received unless stated otherwise. High performance liquid chromatography-grade water was used to prepare all solutions and buffers.

^64^Cu was produced from ^64^Ni(p,n)^64^Cu nuclear reaction on enriched ^64^Ni on a TR-19 biomedical cyclotron (Advanced Cyclotron Systems, Inc.) at Washington University (St. Louis, MO), followed by an automated system using standard procedures for purification.^22,23^ The resulting activity was diluted in 0.1 M hydrochloric acid (HCl), and specific activity ranged from 3.7 to 55.5 GBq/*μ*g (100-1500 mCi/*μ*g). A stock solution of [^64^Cu]CuCl_2_ in 0.1 M HCl was diluted 10-fold with 0.1 M ammonium acetate (NH_4_OAc) pH 5.5, and 148 MBq (4 mCi) was transferred to a microtube. The volume was increased to 50 *μ*L with 0.1 M NH_4_OAc pH 6.0, and an aliquot of 8 *μ*L of *α*_v_*β*_6_-BP (8 nmol) was added. The reaction mixture was incubated at 95°C for 15 minutes. The product was evaluated for radiochemical purity by radio-TLC with a mobile phase of 50 mM diethylenetriamine pentaacetate pH 6.0. The [^64^Cu]Cu-*α*_v_*β*_6_-BP remained at the origin, whereas free [^64^Cu]CuCl_2_ moved with the solvent front.

### Small animal PET/CT imaging and standard uptake value analysis

Mice were injected intravenously with ~11.1 MBq (300 *μ*Ci) of [^64^Cu]Cu-*α*_v_*β*_6_-BP and imaged with CT followed by static PET scans at 1 hour after radiotracer administration on a nanoScan PET/CT scanner (Mediso Medical Imaging Systems). Static images were collected for 45 minutes and reconstructed with expectation maximization algorithm with the Mediso software (Mediso Medical Imaging Systems). Regions of interest (ROI) were selected based on co-registered anatomic CT images, and the radioactivity associated with the lung was measured using the Inveon Research Workstation (Siemens Medical Solutions). In the whole lung RIPF model, the entire irradiated lung was selected as the ROI, whereas in the focal RIPF model, the region with abnormal CT density was selected as the ROI on the co-registered PET image. The mean standard uptake value (SUV mean) was calculated. The NAVPNLRGDLQVLAQKVART peptide was used as a blocking agent.

### Histological analysis and immunohistochemistry

The lungs were harvested and fixed in neutral buffered formalin with the lungs inflated through cannulization. After fixation and dehydration, tissue samples were cut in half to obtain coronal sections close to the isocenter and embedded in paraffin. Tissue sections (5 mm) were obtained for further processing. Masson’s trichrome staining was performed to evaluate collagen deposition within the irradiated lung tissues. In addition, immunohistochemistry (IHC) for *α*_v_*β*_6_ expression was performed using the following protocol. Slides were baked at 55°C for 60 minutes followed by xylene and ethanol wash to deparaffinize and rehydrate the slides. Heat-induced antigen retrieval was performed in citrate buffer 0.1 M pH 6.0 at 92°C for 20 minutes. Tissues were blocked with Dako Endogenous Enzyme block (Dako) for 10 minutes, followed by 10% goat serum in phosphate buffered saline for 45 minutes. A primary anti-*α*_v_*β*_6_ rabbit monoclonal antibody, clone EM05201 (Sigma-Aldrich) at a 1:40 dilution was applied to the slides for incubation overnight at 4°C. The secondary antibody ImmPRESS Goat anti-rabbit (Vector Laboratories Inc) was applied for 45 minutes. Finally, DAB substrate chromogen (Dako) was added, and sections were counterstained with hematoxylin. Sections were dehydrated and mounted with a coverslip.

Slides were digitized using the Zeiss Axioscan 7 Slide Scanner and images were analyzed using Zeiss Zen 3.8 and ImageJ. For the focal RIPF model, the area of fibrosis was measured with the Zeiss Zen area measurement tool. For the whole lung RIPF model, given the diffuse staining, the percent area of fibrosis (based on trichrome staining) was quantified using ImageJ with a threshold-based algorithm as described previously^24^; the percent-stained area was similarly quantified on IHC slides for *α*_v_*β*_6_.

### Statistical analysis

Statistical analysis was performed using GraphPad Prism (version 10.0.2). The SUV mean between the irradiated and sham groups was compared using Welch *t* test. A *P* value < .05 was considered statistically significant.

## Results

### Integrin α_v_β_6_-targeted PET imaging in the focal RIPF model

**Fig. 1.**
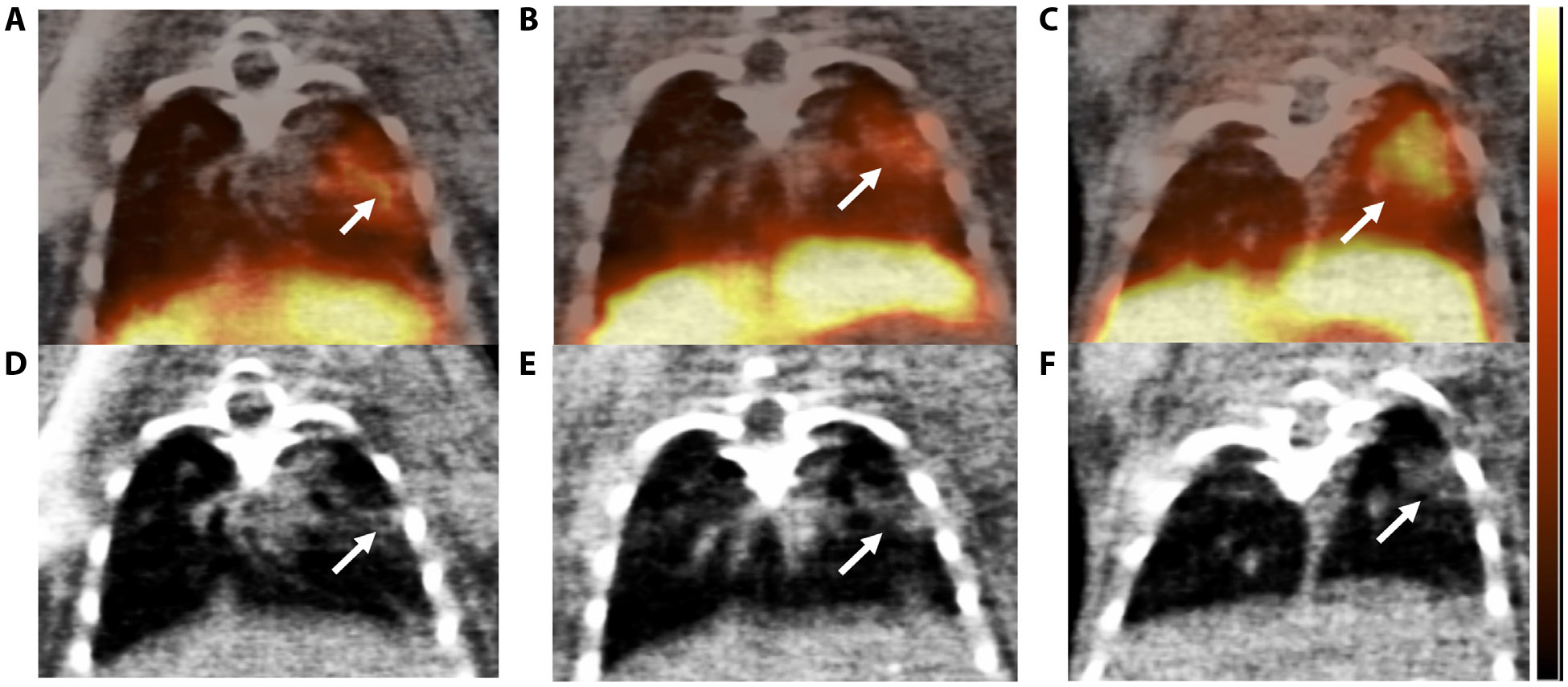
Representative PET/CT images for the focal ablative RIPF model up to 8 weeks post-RT. Images were acquired at 1 hour postinjection of 11.1 MBq of [^64^Cu]Cu-*α*_*v*_*β*_6_-BP at 4 to 8 weeks post-RT (70 Gy delivered focally to a 3 × 3 mm spot in the left lung using an AP:PA configuration). The upper panels (A-C) correspond to PET/CT fusion images (arrows indicate the uptake region), and the lower panels (D-F) show CT images only (arrows indicate the damaged area). Uptake was observed at week 4 (A); the CT image indicated lung tissue damage (D). At week 6, a more diffuse uptake was noticed by PET (B), whereas CT revealed similar lung tissue damage (E). At week 8, there was a more intense and diffuse uptake of [^64^Cu]Cu-*α*_*v*_*β*_6_-BP (C), which was validated by the larger and more diffuse area of lung tissue damage by CT (F). The color bar on the right side represents the SUV mean of 0-0.5. *Abbreviations:* PET/CT = positron emission tomography/computed tomography; RT = radiation therapy; SUV = standard uptake value.

**Fig. 2.**
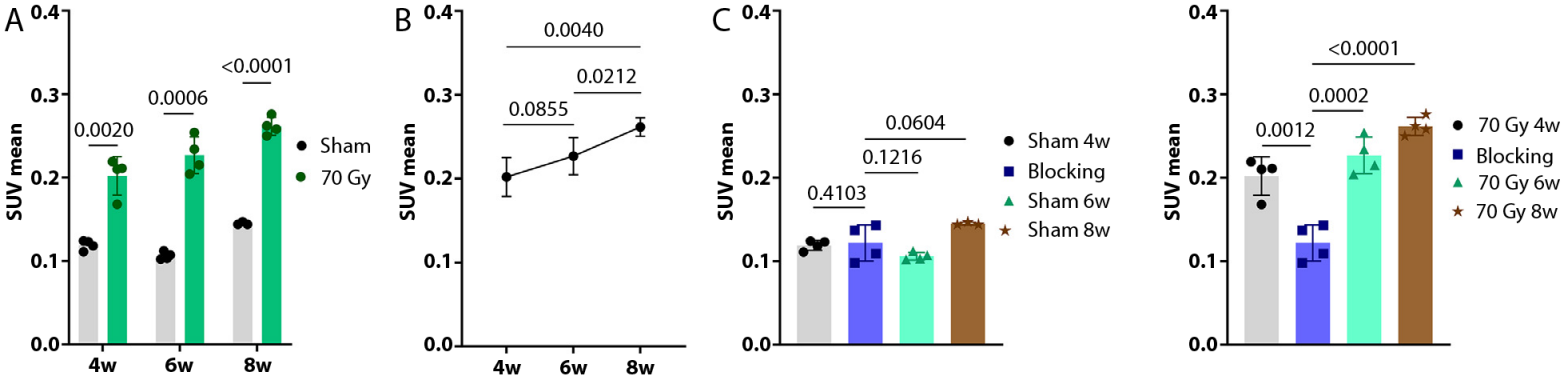
SUV analysis as a function of time in the focal ablative RIPF model shows a progressive increase in uptake of [^64^Cu]Cu-*α*_*v*_*β*_6_-BP from weeks 4 to 8 post-RT. SUV mean comparison demonstrating increased uptake of [^64^Cu]Cu-*α*_*v*_*β*_6_-BP in the irradiated region compared with sham-treated group at week 4 (*P* = .0020), week 6 (*P* = .0006), and week 8 (*P* < .0001) (A). There was a progressive increase in SUV mean as a function of time post-RT from weeks 4 to 6 (P = .0855), 6 to 8 (*P* = .0212), and 4 to 8 (*P* = .0040) in the focal ablative RIPF model (70 Gy delivered to the left lung) (B). Comparison of SUV mean in a blocking experiment at week 5 showed no statistical difference between the blocking group and the sham animals imaged at weeks 4, 6, and 8 (C), but significant decrease in SUV mean in the blocking group vs irradiated animals at week 4, 6, and 8 (D); n = 4 for each group. *Abbreviations:* RIPF = radiation-induced pulmonary fibrosis; SUV, standard uptake value.

**Fig. 3.**
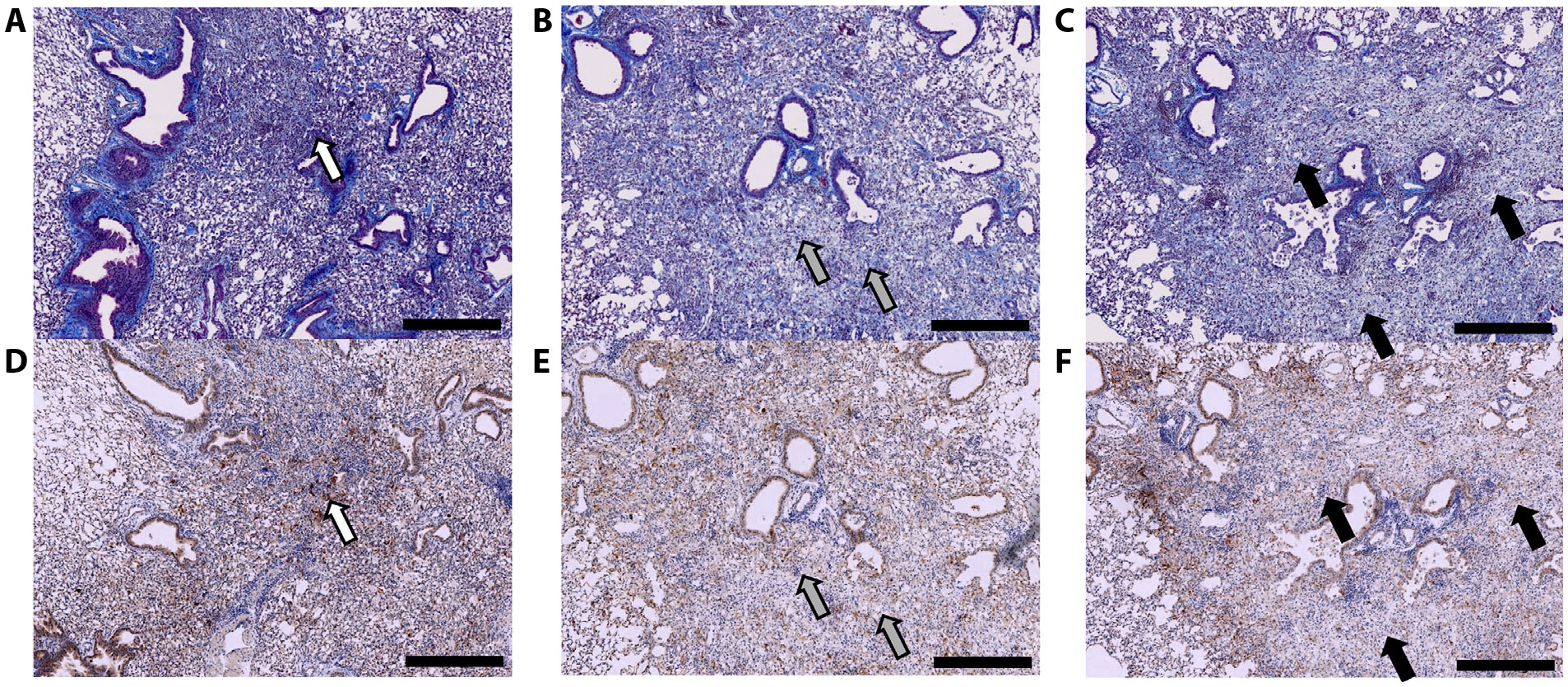
Upregulation of integrin *α*_v_*β*_6_ in the focal ablative RIPF model and corresponding fibrosis development from weeks 4 to 8 post-RT. Trichrome staining (A-C) and corresponding IHC (D-F) of integrin *α*v*β*6 expression at week 4 (A, D), week 6 (B, E), and week 8 (C, F), respectively. Within the irradiated region in the left lung, there was minimal collagen deposition (with the corresponding region showing minimal integrin *α*v*β*6 expression) but dense inflammatory infiltrates (purple nuclei) at week 4 (white arrows; A, D), progressive increase in collagen deposition and integrin *α*v*β*6 expression at week 6 (gray arrows; B, E), and significant collagen deposition by week 8 (black arrows; C, F). The region of fibrosis increased from 2.7 mm^2^ at week 4, to 4.3 mm^2^ at week 6, and to 5.7 mm^2^ at week 8. Scale bar = 500 microns. *Abbreviations:* IHC, immunohistochemistry; RIPF = radiation-induced pulmonary fibrosis.

In the focal RIPF model, there was increased uptake of [^64^Cu]Cu-*α*_v_*β*_6_-BP in the irradiated region, compared with the unirradiated contralateral lung, as demonstrated in Figure 1 showing representative small animal PET/CT fusion images at 4, 6, and 8 weeks post-RT (70 Gy delivered focally to the left lung; Fig. E3). The region of increased focal uptake of [^64^Cu]Cu-*α*_v_*β*_6_-BP corresponded to the region with increased density in conventional CT images. At week 4, the SUV mean of the irradiated group was higher than the sham group (0.202 ± 0.023 vs 0.119 ± 0.006, *P* = .0020). At week 6, the SUV mean continued to increase, compared with the sham group (0.227 ± 0.022 vs 0.106 ± 0.005, *P* = .0006). At week 8, the SUV mean was significantly higher in the irradiated group compared with the sham group (0.262 ± 0.011 vs 0.145 ± 0.002, *P* < .0001) (Fig. 2A). Of note, there was a progressive increase in the SUV mean from week 4 to 8 (Fig. 2B). Although the increase in SUV mean between weeks 4 and 6 was not statistically significant (*P* =.0855), the increase in uptake of [^64^Cu]Cu-*α*_v_*β*_6_-BP from week 6 to 8 and from week 4 to 8 was statistically significant (*P* =.0212 and *P* = .0040, respectively). Histologic correlation with hematoxylin and eosin and trichrome staining was also performed at each time point from week 4 to 8 in the focal RIPF model. Trichrome staining at 4 weeks demonstrated minimal collagen deposition (blue staining), although at this time point there was substantial inflammation, visualized as purple inflammatory cell nuclei (Fig. 3A). There was a small focus of integrin *α*_v_*β*_6_ expression within the irradiated region at 4 weeks (Fig. 3D), with progressive increases in collagen deposition and integrin *α*_v_*β*_6_ expression at week 6 (Fig. 3B, E). At week 8, there was a significant amount of collagen observed within the irradiated region, corresponding to the region with increased integrin *α*_v_*β*_6_ expression (Fig. 3C, F). The region of fibrosis was 2.7 mm^2^ at week 4 and progressively increased to 4.3 mm^2^ at week 6 and 5.7 mm^2^ at week 8. This is consistent with the small animal PET/CT imaging findings, which suggest a progressive increase in *α*_v_*β*_6_ expression from week 4 to week 8 (Fig. 2).

A blocking experiment demonstrated specificity of [^64^Cu]Cu-*α*_v_*β*_6_-BP for integrin *α*_v_*β*_6_. The SUV mean between the blocking group and sham animals at weeks 4 and 6 were similar (*P* = .4103, and *P* = .1216, respectively) (Fig. 2C). Likewise, the blocking agent led to a significant reduction in radiotracer uptake, compared with the irradiated animals at week 4 (P = .0012) and week 6 (P = .0002), respectively (Fig. 2D).

### Integrin α_v_β_6_-targeted PET imaging in the whole lung RIPF model

**Fig. 4.**
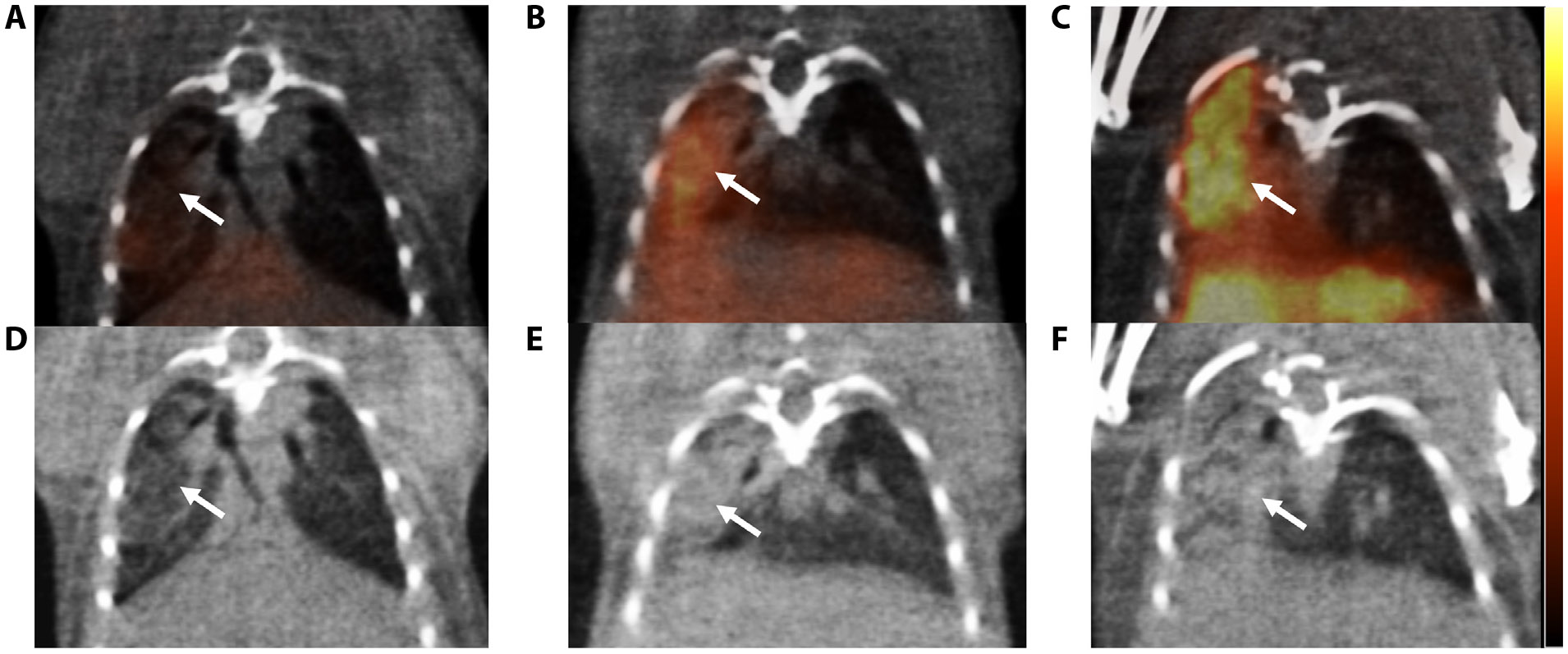
Representative PET/CT images demonstrating increased [^64^Cu]Cu-*α*_*v*_*β*_6_-BP uptake in the whole lung RIPF model from 4 to 6 months post-RT. Mice received 14 Gy delivered with a 10 × 10 mm collimator to the right lung using an AP:PA configuration) from months 4 to 6, acquired 1 hour postinjection of 11.1 MBq of [^64^Cu]Cu-*α*_*v*_*β*_6_-BP. The upper panel (A-C) shows the PET/CT fusion images (white arrows indicate the region with uptake), and the lower panel (D-F) shows the CT images only (arrows point to the corresponding area). Minimal uptake was observed at month 4 (A); the corresponding CT image shows mildly increased density in the right lung (D). At month 5, there was moderate uptake of the radioligand (B), with significantly increased CT density in the right lung (E). At month 6, there was more intense and diffuse uptake of [^64^Cu]Cu-*α*_*v*_*β*_6_-BP (C), with diffuse opacification of the right lung on CT (F). The color bar represents an SUV of 0 to 0.5; n=4 each group. *Abbreviations:* PET/CT = positron emission tomography/computed tomography; RIPF = radiation-induced pulmonary fibrosis; RT = radiation therapy; SUV = standard uptake value.

**Fig. 5.**
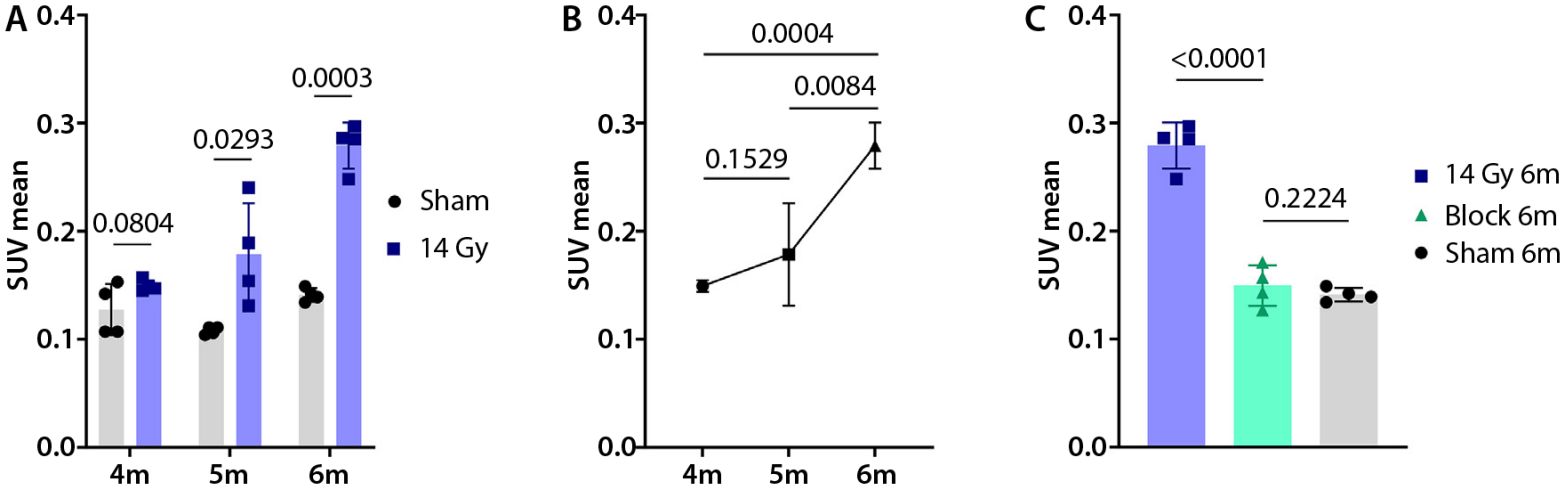
SUV analysis in the whole lung RIPF model shows increased [^64^Cu]Cu-*α*_*v*_*β*_6_-BP activity in the whole lung RIPF model from 4 to 6 months post-RT. SUV mean as a function of time post-RT from months 4 to 6 in the whole lung RIPF model (14 Gy delivered to the right lung), demonstrating similar uptake to sham (*P* = .0804) at month 4, moderate increase in SUV at month 5 (*P* = .0293), and significant increase in SUV at month 6 (*P* = .0003), compared with the sham group (A). SUV mean plotted as a function of time between months 4 and 6, showing no significant increase in uptake between months 4 and 5(*P* = .1529), but upregulation of *α*_*v*_*β*_6_ between months 5 and 6 (*P* = .0084) and months 4 and 6 (*P* = .0004) (B). Comparison of SUV mean in a blocking experiment at month 6 showed a significant reduction in uptake in the blocking group compared with the irradiated group at month 6 without blocking (*P* < .0001), and no statistical difference between the blocking group and the sham animals imaged at month 6 (*P* = .2224) (C), demonstrating the specificity of the radiotracer; n = 4 for each group. *Abbreviations:* RIPF = radiation-induced pulmonary fibrosis; RT = radiation therapy; SUV = standard uptake value.

We further studied RIPF development in a whole lung RIPF model by delivering 14 Gy to the right lung of C57BL/6J mice. Figure 4 shows a representative set of PET/CT fusion images from months 4 to 6 in the whole lung RIPF model. At month 4, [^64^Cu]Cu-*α*_v_*β*_6_-BP uptake in the irradiated lung was similar to sham (0.149 ± 0.005 vs 0.127 § 0.024, *P* = .0804). However, at month 5, we observed a significant difference in SUV mean between the irradiated lung and sham (0.179 ± 0.047 vs 0.108 ± 0.004, *P* = .0293). At month 6, the difference between the irradiated lung and sham was more pronounced (0.279 ± 0.021 vs 0.141 ± 0.006, P = .0003) (Fig. 5A).

In addition, we observed a nonsignificant increase in SUV mean between months 4 and 5 (*P* = .1529). However, there was a significant increase in uptake of the radiotracer from month 5 to 6 (*P* = .0084), and from months 4 to 6 (*P* = .0004) (Fig. 5B). The blocking experiment conducted at the 6 month time point revealed a significant reduction in uptake of the radiotracer in the blocked group compared with the irradiated group (*P* < .0001). We observed a similar SUV mean between the blocked group and the sham group (*P* = .2224) (Fig. 5C).

**Fig. 6.**
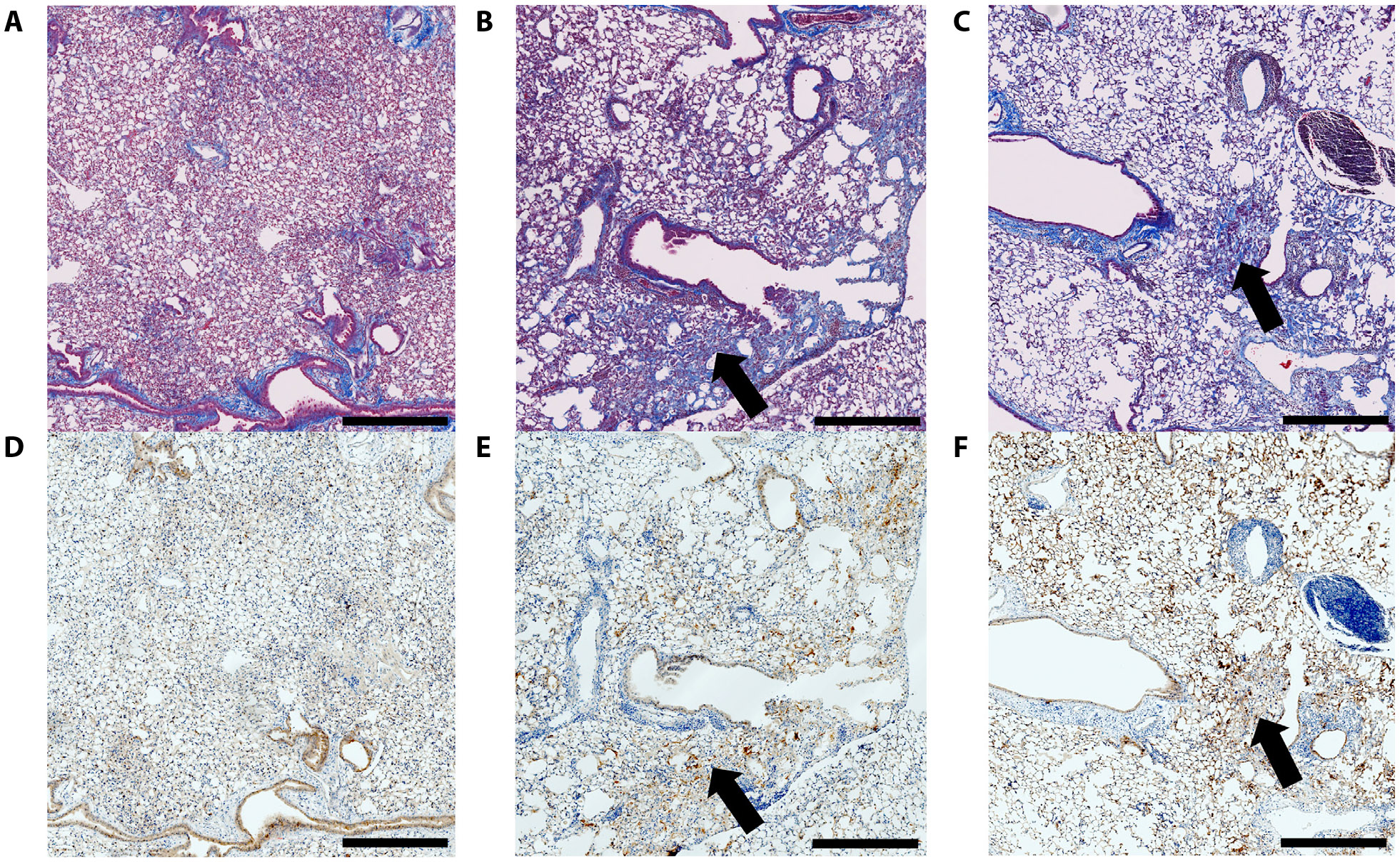
Trichrome and IHC staining demonstrating gradual upregulation of integrin *α*_v_*β*_6_ in the whole lung RIPF model and corresponding development of fibrosis from months 4 to 6. Histologic analysis of the whole lung RIPF model at months 4, 5, and 6, respectively. Masson’s trichrome staining (A-C) at months 4, 5, and 6, respectively, with corresponding IHC (D-F) demonstrating minimal *α*_*v*_*β*6 expression at month 4 (1.5% of the total area) and progressive increase at month 5 (2.0% of the total area) and more diffuse expression at month 6 throughout the right lung (4.8% of the total area). Areas with fibrosis have correspondingly high *α*_*v*_*β*_6_ expression (indicated by arrows). The percent area of fibrosis was 5.1% at month 4, 10.1% at month 5, and 19.6% at month 6. Scale bar = 500 microns. *Abbreviations:* IHC = immunohistochemistry; RIPF = radiation-induced pulmonary fibrosis.

Histological analysis of the irradiated right lung from months 4 to 6 showed that there was minimal integrin *α*_v_*β*_6_ expression 4 months post-RT (1.5% of total area) and minimal collagen deposition (5.1% of total area) within the right lung (Fig. 6A, D), and increased integrin *α*_v_*β*_6_ expression (2.0% of total area) 5 months post-RT in the irradiated lung with focal islands of fibrosis and the percent area of fibrosis increased to 10.1% (Fig. 6B, E). At month 6 post-RT, there was prominent and diffuse integrin *α*_v_*β*_6_ expression (4.8% of total area) throughout the irradiated right lung and diffuse deposition of collagen in the right lung (19.6% of total area), in addition to scattered regions of fibrosis (corresponding to areas with significant integrin *α*_v_*β*_6_ expression) (Fig. 6C, F).

## Discussion

In this study, we demonstrated that longitudinal integrin *α*_v_*β*_6_-targeted PET imaging with [^64^Cu]Cu-*α*_v_*β*_6_-BP can detect RIPF at an early stage in both a focal high-dose RIPF model and a whole lung RIPF model. Integrin *α*_v_*β*_6_ is an attractive target to study the evolution of RIPF in vivo, given its central role in the pathogenesis of RIPF. In particular, integrin *α*_v_*β*_6_, which is expressed at a low level in the normal alveolar epithelium, is upregulated upon radiation injury, leading to latent TGF-*β*1 activation.^25^ Therefore, it serves as an important early molecular biomarker for the development of fibrosis.

In the high-dose RIPF model, the region of increased uptake of [^64^Cu]Cu-*α*_v_*β*_6_-BP corresponded well to the focally irradiated region with increased density on CT (Fig. 1). In addition, this correlated to the area of fibrosis as validated histologically with trichrome staining to assess collagen and IHC for integrin *α*_v_*β*_6_ expression. The timing for the development of fibrosis was consistent with a prior study that demonstrated the use of focal ablative doses of RT in a murine model.^20,21^

Compared with the focal RIPF model, we observed a more gradual onset of integrin *α*_v_*β*_6_ expression and subsequent development of fibrosis in the whole lung RIPF model. Here, [^64^Cu]Cu-*α*_v_*β*_6_-BP uptake was not significant at month 4 post-RT after delivering 14 Gy to one lung, and IHC for integrin *α*_v_*β*_6_ expression confirmed the findings. There was a correspondingly low amount of collagen deposition at this time point. However, at month 5, increased uptake of ^64^[Cu]Cu-*α*_v_*β*_6_-BP was observed, as confirmed by IHC for integrin avb6. There were also discrete, small islands of fibrosis in the irradiated lung (Fig. 6B, E). By month 6 post-RT, there was diffuse and significantly increased uptake of [^64^Cu]Cu-*α*_v_*β*_6_-BP in the entire irradiated lung. There was correspondingly diffuse heterogeneous deposition of collagen interstitially, in addition to focal islands of fibrosis with particularly high integrin *α*_v_*β*_6_ expression (Fig. 6C, F). The timing of onset of integrin *α*_v_*β*_6_ expression observed here was consistent with an earlier study, which demonstrated that integrin *α*_v_*β*_6_ expression remained at a low level until 18–20 weeks after 14 Gy was delivered to the thorax when expression markedly increased around areas that later developed fibrosis.^5^

Here, we showed that there was minimal expression of integrin *α*_v_*β*_6_ in healthy lung tissue before the development of fibrosis, indicating the feasibility of detecting RIPF at an early stage using this imaging probe. Other groups have used integrin *α*_v_*β*_6_–targeted PET and SPECT imaging to detect idiopathic pulmonary fibrosis using cystine-knot peptide,^16,26^ [^18^F]F-A20FMDV2,^6^ and vimentin-targeting peptide (for SPECT imaging),^27^ whereas our work focused on imaging integrin *α*_v_*β*_6_ expression in an RIPF model. It was interesting to see that the onset of integrin *α*_v_*β*_6_ expression was significantly delayed in the whole lung RIPF model, but in both models, the region of increased integrin *α*_v_*β*_6_ expression correlated well with the area of fibrosis. The biological insights obtained in both models are valuable in investigating strategies to mitigate the development of RIPF at an early stage, by targeting therapeutic administration during the integrin avb6 upregulation to inhibit its development. A limitation of the current study is that integrin *α*_v_*β*_6_ is also expressed highly in tumors, as shown in prior studies investigating the use of integrin *α*_v_*β*_6_ targeted PET tracers to image pancreatic, cervical, and lung cancers,^14,16^ which may complicate the detection of radiation-induced fibrosis from recurrent disease in the clinical setting. However, this is readily addressable by subtracting the radiation target volume, allowing for independent interpretation of the PET tracer expression in nontargeted lungs, as has been successfully performed in other studies.^28^

We have focused primarily on the lung in this study, whereas several prior studies showed the important role that integrin *α*_v_*β*_6_ plays in the development of fibrosis in a number of models of fibrosis, such as those in the heart, liver, pancreas, kidney, and biliary tract.^11,25,29-33^ In addition, inhibition of integrin *α*_v_*β*_6_ has been shown to be an effective therapeutic approach to prevent the subsequent development of fibrosis.^5,7,34^ Therefore, integrin *α*_v_*β*_6_-targeted PET imaging can also serve as an important tool for therapeutic discovery to assess response to novel integrin-targeted therapies to prevent fibrosis in many organs.

## Conclusions

In this study, we demonstrated the utility of integrin *α*_v_*β*_6_–targeted PET imaging with [^64^Cu]Cu-*α*_v_*β*_6_-BP to monitor the development of RIPF in a focal high-dose RIPF model and a whole lung RIPF model. Integrin *α*_v_*β*_6_ is a promising early molecular imaging biomarker that has a central role in the pathogenesis of fibrosis, and its expression corresponds well with the development of RIPF. Integrin *α*_v_*β*_6_-targeted PET imaging can serve as a useful tool to study the evolution of RIPF in vivo, with significant clinical implications.

## Supplementary Material

SuppFig_final

Supplementary material associated with this article can be found in the online version at doi:10.1016/j.ijrobp.2024.08.034.

## Data Availability

Research data are stored in an institutional repository and will be shared on request to the corresponding author.
