# Supplementary material for: Using Integrin αvβ6-Targeted Positron Emission Tomography Imaging to Longitudinally Monitor Radiation-Induced Pulmonary Fibrosis In Vivo
Source: Int J Radiat Oncol Biol Phys. Author manuscript; Available in PMC 2025 Jul 7. (PMC12232630; doi:10.1016/j.ijrobp.2024.08.034)
Supplement: SuppFig_final [file NIHMS2091100-supplement-SuppFig_final.pptx]

## Slide 1
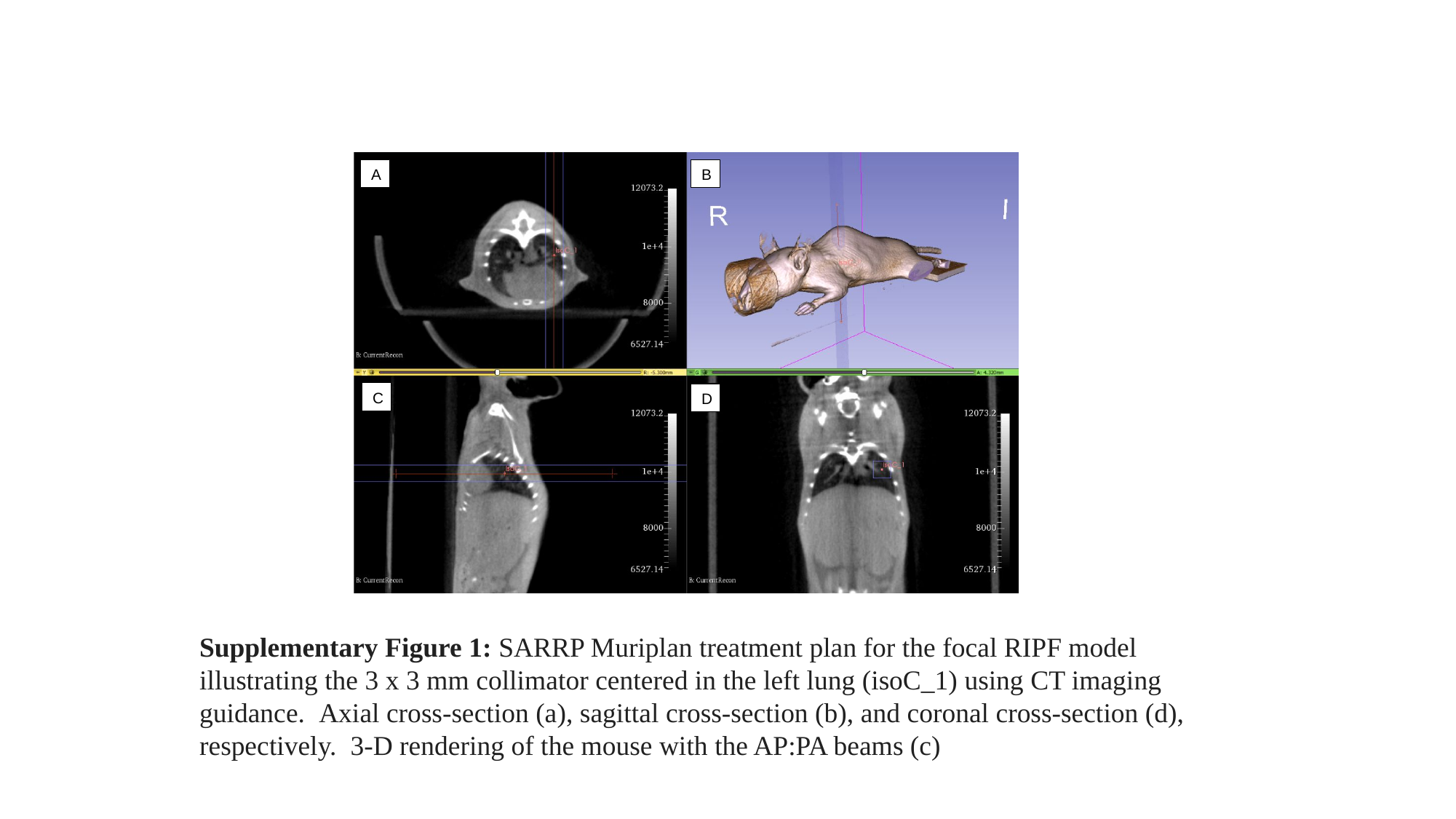

A
B
C
D
Supplementary Figure 1: SARRP Muriplan treatment plan for the focal RIPF model illustrating the 3 x 3 mm collimator centered in the left lung (isoC_1) using CT imaging guidance. Axial cross-section (a), sagittal cross-section (b), and coronal cross-section (d), respectively. 3-D rendering of the mouse with the AP:PA beams (c)

## Slide 2
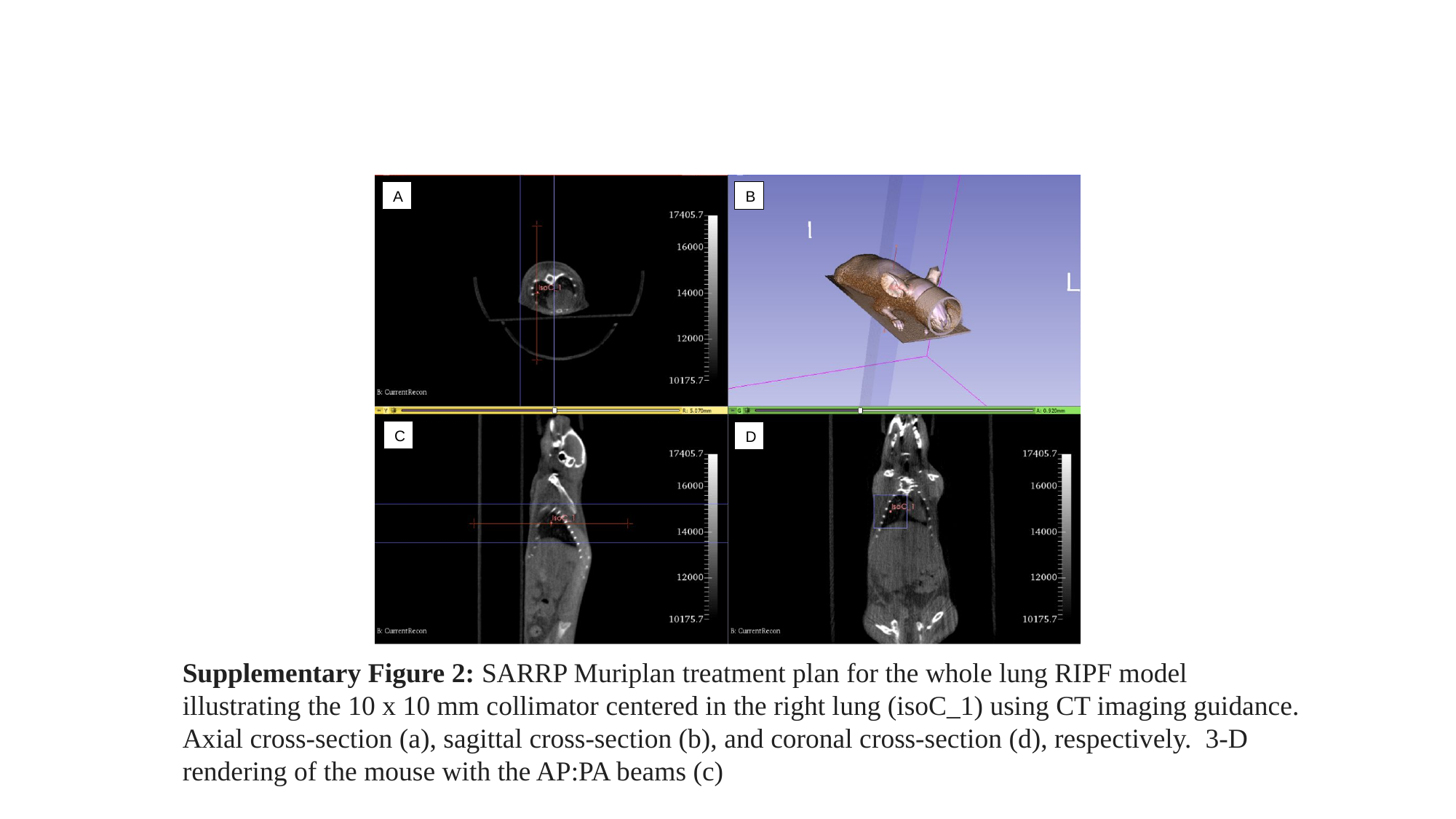

A
B
C
D
Supplementary Figure 2: SARRP Muriplan treatment plan for the whole lung RIPF model illustrating the 10 x 10 mm collimator centered in the right lung (isoC_1) using CT imaging guidance. Axial cross-section (a), sagittal cross-section (b), and coronal cross-section (d), respectively. 3-D rendering of the mouse with the AP:PA beams (c)

## Slide 3
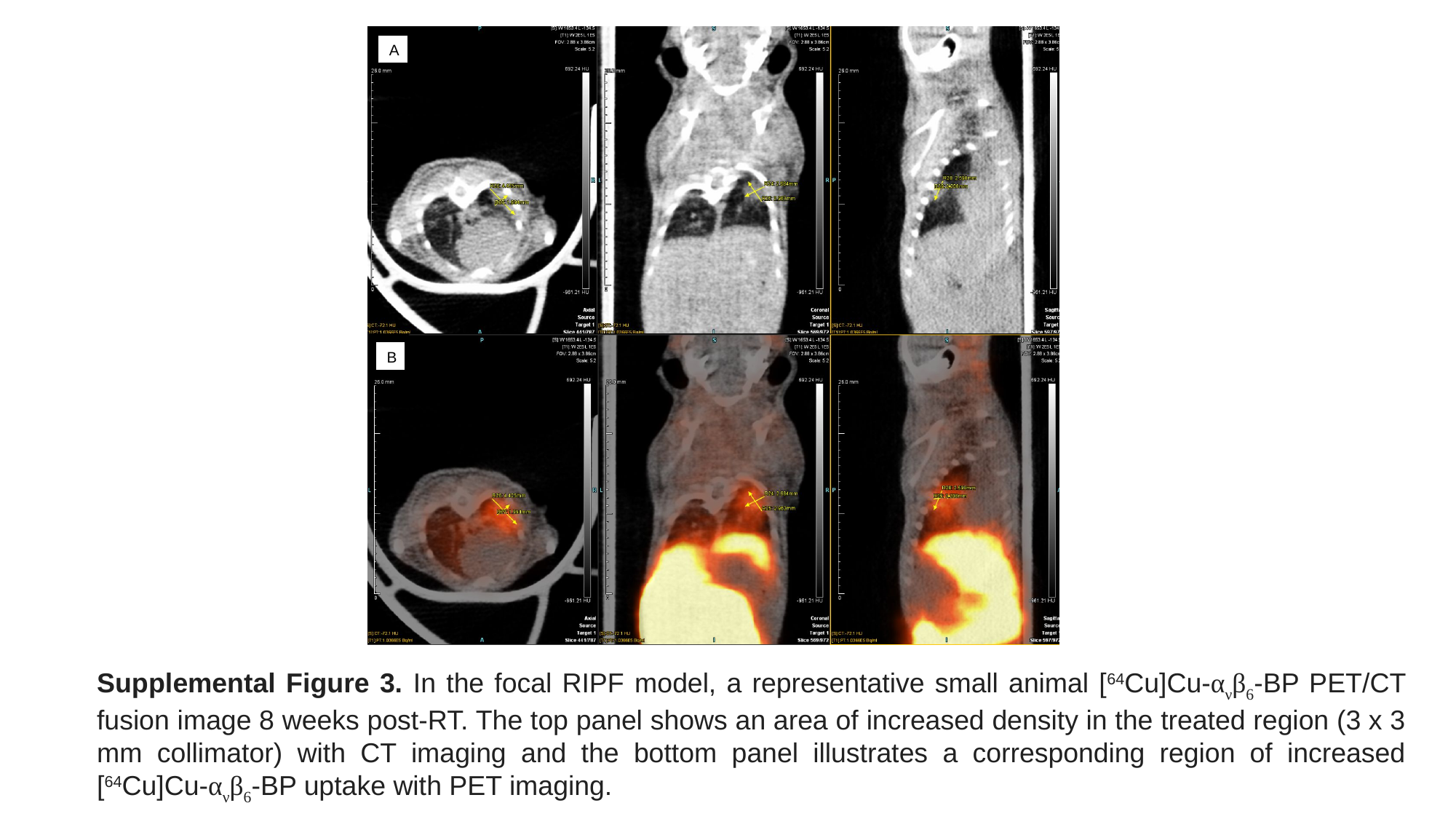

A
B
Supplemental Figure 3. In the focal RIPF model, a representative small animal [64Cu]Cu-ανβ6-BP PET/CT fusion image 8 weeks post-RT. The top panel shows an area of increased density in the treated region (3 x 3 mm collimator) with CT imaging and the bottom panel illustrates a corresponding region of increased [64Cu]Cu-ανβ6-BP uptake with PET imaging.
